# A Two-Ply Polymer-Based Flexible Tactile Sensor Sheet Using Electric Capacitance

**DOI:** 10.3390/s140202225

**Published:** 2014-01-29

**Authors:** Shijie Guo, Takahisa Shiraoka, Seisho Inada, Toshiharu Mukai

**Affiliations:** 1 Tokai Rubber Industries, Ltd., 1 Higashi, 3-chome, Komaki-shi, Aichi-ken 485-8550, Japan; E-Mails: takahisa.shiraoka@tri.tokai.co.jp (T.S.); seisho.inada@tri.tokai.co.jp (S.I.); 2 RIKEN-TRI Collaboration Center for Human-Interactive Robot Research, RIKEN, 2271-130, Anagahora, Shimoshidami, Moriyama-ku, Nagoya 463-0003, Japan; E-Mail: tosh@nagoya.riken.jp

**Keywords:** capacitive tactile sensor, tactile sensor, flexible sensor, polymer, conductive rubber, screen printing

## Abstract

Traditional capacitive tactile sensor sheets usually have a three-layered structure, with a dielectric layer sandwiched by two electrode layers. Each electrode layer has a number of parallel ribbon-like electrodes. The electrodes on the two electrode layers are oriented orthogonally and each crossing point of the two perpendicular electrode arrays makes up a capacitive sensor cell on the sheet. It is well known that compatibility between measuring precision and resolution is difficult, since decreasing the width of the electrodes is required to obtain a high resolution, however, this may lead to reduction of the area of the sensor cells, and as a result, lead to a low Signal/Noise (S/N) ratio. To overcome this problem, a new multilayered structure and related calculation procedure are proposed. This new structure stacks two or more sensor sheets with shifts in position. Both a high precision and a high resolution can be obtained by combining the signals of the stacked sensor sheets. Trial production was made and the effect was confirmed.

## 1. Introduction

With the practical use of nursing care assistance robots and living assistance robots as well as various human-interactive care and medical machines in recent years, the demands on large-area flexible tactile sensors as interfaces with humans are increasing. To meet the demands, various tactile sensors have been proposed, studied, and developed. Such sensors include tactile sensors that employ discrete semiconductors [1], contact resistance [2,3], conductive rubber [4–6], piezoelectric polymers [7], and electric capacitance [8,9]. Our research group has carried out research and development on tactile sensors by integrating semiconductor sensors and mounting them on the soft exterior of the care assistance Robot for Interactive Body Assistance (RIBA) [10–12]. This enabled the operation of the robot based on the human sense of touch and the detection of contact pressure with the person lifted by the robot. However, it is practically difficult to cover the entire body of the robot with such tactile sensors because semiconductor sensors are expensive. Sensor sheets that employ contact resistance and piezoelectric polymers have bending flexibility, but low compatibility with humans; it is necessary for humans to feel that the sensor sheets are pleasant to touch, a property exhibited by cloth and rubber. Also, fashioning such sensor sheets into complicated shapes is difficult.

**Figure 1.**
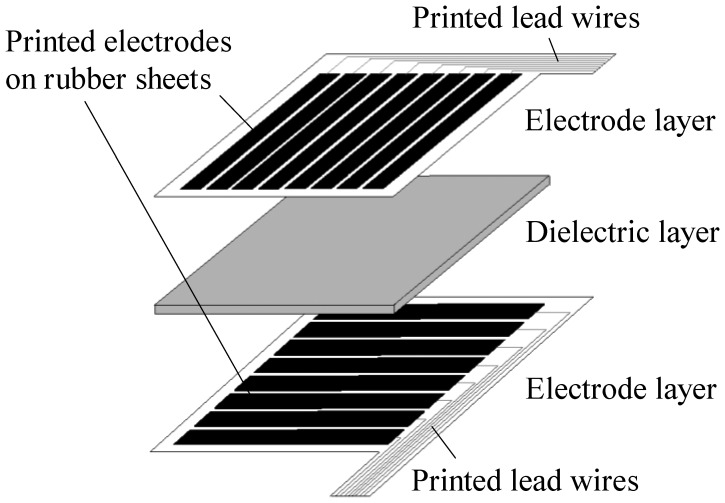
Schematic structure of a traditional capacitive tactile sensor sheet.

To solve the problems of the above-mentioned sensors, our research group has developed capacitive soft sensor sheets made entirely of polymers such as rubber and urethane foam without the use of metal parts, and applied them to the newly developed care assistance robot RIBA-II [13], a successor of RIBA. Figure 1 shows the schematic of such a capacitive tactile sensor sheet. It has a three-layered structure, with a dielectric layer sandwiched by two electrode layers. Each electrode layer has a number of parallel ribbon-like electrodes. The electrodes on the two electrode layers are oriented orthogonally and each crossing area of two perpendicular electrodes makes up a capacitive sensor cell on the sheet. The sheet is an assembly of discretely and independently distributed sensor cells. To avoid confusion, in this paper we call the structure shown in Figure 1 a traditional sensor sheet. Such a structure has been proposed previously and sensor sheets that employ metal electrodes are commercially available [14]. In the applications to care-related machines, including care assistance robots, however, metal electrodes still have problems such as their low stretchability and high cost. We have devised a method of forming electrode layers by screen-printing conductive rubber onto a flexible rubber sheet to realize compatibility with humans at a low manufacturing cost. This method can also be applied to sensors with a complicated shape and is suitable for the fabrication of sensors on large-area substrates at a low cost.

Considering practical use, it is desirable to measure a pressure at both a high precision and a high resolution, but it is difficult for the structure shown in Figure 1 to meet both objectives simultaneously. The resolution of the sensor sheet is the area of a cell and decreasing the width of the electrodes and the gap between two adjacent electrodes to obtain a high resolution is inevitable. On the other hand, soft and high elastic polymer materials such as urethane foam or rubber are used as a dielectric layer to have a high flexibility. These materials usually have low electric permittivity, so decreasing the width of the electrodes implies decreasing the area of a cell and this in turn results in a low capacitance under a certain pressure. As a small capacitance is more easily affected by the electric noises from the lead wires and the circuit boards, decreasing electrode width makes it difficult to measure a small pressure at a high Signal/Noise (S/N) ratio and consequently leads to more complicated and large-scale electronic circuits and higher manufacturing costs, *i.e.*, compatibility between precision and resolution is difficult. To overcome this problem, a new multilayered structure is proposed. This new structure stacks two or more sensor sheets with shifts in position. Both a high precision and a high resolution can be obtained by combining the signals of the stacked sensor sheets. This paper describes the proposed two-ply structure and the related calculation procedure, and furthermore, reports the results of trial production and experiments.

## 2. A Traditional Sensor Sheet and Its Problems

### 2.1. The Structure and Principle of a Traditional Sensor Sheet

As shown in Figure 1, the structure of a traditional capacitive tactile sensor sheet is simple: a thin dielectric layer is sandwiched by two electrode layers. Each electrode layer has a number of parallel electrodes. The electrodes on the two layers are oriented orthogonally to each other, so that independent capacitive sensor cells are formed by the intersection of the two orthogonal electrode layers. When the numbers of electrodes in the upper and lower layers are *M* and *N*, respectively, *M* × *N* capacitive sensor cells are formed on a sensor sheet.

The capacitance of the cell formed by the intersection of the *i*th electrode of one electrode layer and the *j*th electrode of the other electrode layer, *C*(*i,j*), is given by:

(1) $$\begin{array}{cc} C(i,j)={ɛ}_{0}{ɛ}_{r}\frac{s(i,j)}{d(i,j)} & \left(i=1,2,\cdots ,M;\;j=1,2,\cdots ,N\right) \end{array}$$

**Figure 2.**
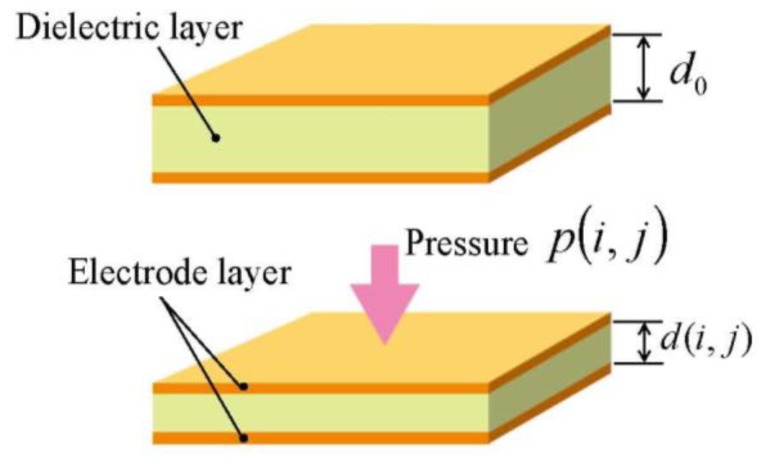
Deformation of a sensor cell subjected to a normal pressure.

Here, *ε*_0_ is the permittivity in vacuum, *ε_r_* is the relative permittivity of the dielectric layer, and *d*(*i, j*) and *s*(*i, j*) are the interelectrode distance (*i.e.*, the thickness of the dielectric layer) and the area of the cell (*i,j*), respectively. Thickness *d*(*i,j*) depends only on the pressure applied on the cell (*i,j*) (see Figure 2). Let Δ*d*(*i,j*) represent the displacement of the cell (*i,j*) in normal direction, *i.e.*, the change of the thickness of the cell (*i,j*), we can express it as:

(2) $$\Delta d(i,j)=d_{0}-d(i,j)$$

Here, *d*_0_ represents the interelectrode distance when no pressure is applied to the cell (*i.e.*, the interelectrode distance before deformation). Since Δ*d*(*i,j*) depends only on the pressure applied, we can express it as a function of pressure *p*(*i,j*):

(3) Δd(i,j)=f[p(i,j)]

Provided *C*(*i,j*) is measured for all cells by switching the electrodes and *d*(*i,j*) is calculated using Equation (1), we can calculate the pressure applied to each cell, *p*(*i,j*), from Equations (2) and (3), as follows:

(4) $$p(i,j)=f^{-1}[\Delta d(i,j)]=f^{-1}[d_{0}-d(i,j)]$$

If the pressure is small and consequently the displacement ΔI(*i,j*) is small enough compared with the undeformed thickness *d*_0_ so that the small deformation theory holds, the relation between pressure and displacement can be expressed as:

(5) $$p(i,j)=Y\frac{\Delta d(i,j)}{d_{0}}=Y\frac{d_{0}-d(i,j)}{d_{0}}$$

Here, *Y* is the Young's modulus of the dielectric layer. Calculating the pressures applied to all *M* × *N* cells will determine the distribution of pressure applied on the sensor sheet. It should be noted that, as shown in Figure 2, it is assumed that the pressure is uniformly applied inside a cell and that the cell is uniformly deformed. Sensor sheets with such a structure are fundamentally designed on the basis of this assumption.

**Figure 3.**
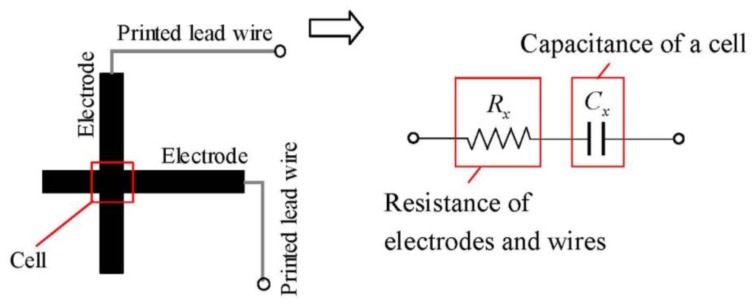
Equivalent circuit of a sensor cell.

**Figure 4.**
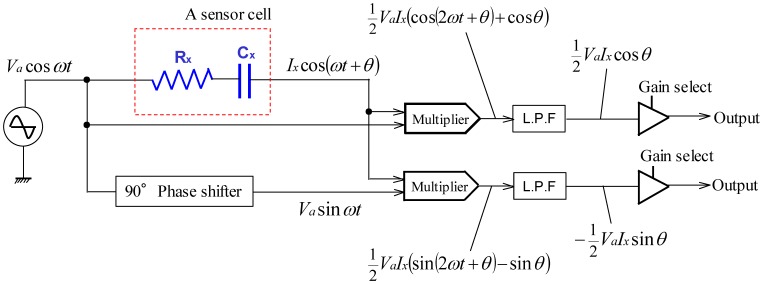
The approach of detecting the resistance and capacitance of a cell.

Figure 3 shows the equivalent circuit of a sensor cell. The quantities to be measured are denoted with a subscript *x* for convenience. In Figure 3, *C_x_* is the capacitance of a cell and *R_x_* is the resistance of the electrodes and wires of the cell. The electrodes also serve as the wires to a cell. Although there are various methods for detecting capacitance, we adopted the impedance vector method since this approach has a high responsiveness and can separate electric resistance *R_x_* and capacitance *C_x_*. Figure 4 shows the flow of the approach. It applies a harmonic voltage:

(6) $$\nu_{a}=V_{a}\cos\omega t$$

to a sensor cell and multiplies it and its 90-degree phase shift by the current flowing through the sensor cell.

Here symbols *ν_a_* and *V_a_* represent the voltage and its amplitude, *t* and ω represent time and frequency, respectively. Let:

(7) $$i_{x}=I_{x}\cos(\omega t+\theta )$$

represent the current flowing through *R_x_* and *C_x_*. Here *i_x_* and *I_x_* represent the current and its amplitude, *θ* represents the phase difference between the current and the applied voltage expressed by Equation (6). Multiply the voltage given in Equation (6) and its 90-dgree phase shift by the current expressed by Equation (7), we get:

(8) $$\begin{array}{ll} u_{r} & =V_{a}\cos(\omega t)\cdot I_{x}\cos(\omega t+\theta )=V_{a}I_{x}(\cos^{2}\;\omega t\;\cos\theta -\cos\omega t\;\sin\omega t\;\sin\theta )\\ & =\frac{1}{2}V_{a}I_{x}\cos\theta +\frac{1}{2}V_{a}I_{x}\cos(2\omega t+\theta ) \end{array}$$

(9) $$\begin{array}{ll} u_{i} & =V_{a}\sin(\omega t)\cdot I_{x}\cos(\omega t+\theta )=V_{a}I_{x}(\sin2\omega t\cos\theta -\sin^{2}\omega t\sin\theta )\\ & =-\frac{1}{2}V_{a}I_{x}\sin\theta +\frac{1}{2}V_{a}I_{x}\sin(2\omega t+\theta ) \end{array}$$

Taking the direct components in Equations (8) and (9) and expressing them as:

(10) $${ū}_{r}=\frac{1}{2}V_{a}I_{x}\cos\theta$$

(11) $${ū}_{i}=\frac{1}{2}V_{a}I_{x}\sin\theta$$

we can calculate *R_x_* and *C_x_* by using the equivalent circuit shown in Figure 3:

(12) $$R_{x}=\frac{1}{2}{V_{a}}^{2}\frac{{ū}_{r}}{{{ū}_{r}}^{2}+{{ū}_{i}}^{2}}$$

(13) $$C_{x}=\frac{2}{{V_{a}}^{2}}\frac{{{ū}_{r}}^{2}+{{ū}_{i}}^{2}}{\omega {ū}_{i}}$$

### 2.2. Problems of a Traditional Sensor Sheet

It is obvious that measurement with a high precision and a high resolution is desirable, but the two issues are not compatible. For the structure shown in Figure 1, the resolution can be increased by decreasing the width of the electrodes and the gap between adjacent electrodes, however, this will reduce the capacitance of a cell and consequently lead to a low S/N ratio. A small capacitance is difficult to measure accurately since it is more easily affected by various electric noises such as those arising from the wires, the circuit boards, and the sensor sheet itself. To remove the noise, more complicated and large-scale electronic circuits are usually required and this will increase the manufacturing costs.

From the relationship expressed by Equation (1), there are three methods of increasing capacitance: increasing the relative permittivity of the dielectric layer, decreasing the thickness of the dielectric layer, and increasing the area of the cells (increasing the width of the electrodes). As is well known, harder materials tend to be required to have a high permittivity and this will result in reduced flexibility. To decrease the thickness of the dielectric layer, the allowable amount of deformation will be reduced, which not only decreases flexibility but also makes it easier for electrode layers to come into contact with each other, resulting in a narrower measurable range. Increasing the width of the electrodes can easily increase capacitance, but it is accompanied by a low resolution. Thus, any of the above methods can increase the capacitance under a certain pressure to get a high measurement precision, but at the cost of lost flexibility or resolution. Therefore, traditional capacitive tactile sensor sheets cannot be used to simultaneously satisfy the three requirements of resolution, measurement precision, and flexibility.

## 3. Proposed Multilayered Structure

### 3.1. Structure of Multilayered Capacitive Tactile Sensor

**Figure 5.**
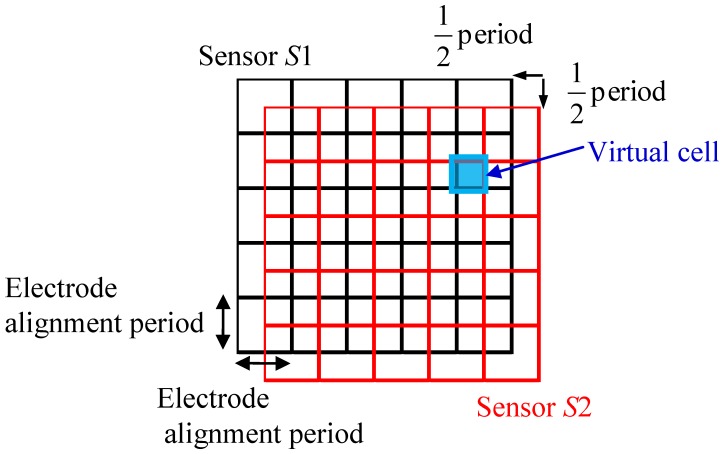
Conceptual diagram of a two-ply sensor sheet made by stacking two traditional sensor sheets.

A sensor with a multilayered structure is proposed to solve the problems mentioned in Section 2.2. Figure 5 shows a conceptual diagram of the structure. It stacks two identical sensor sheets S1 and S2 with a shift in position. The shift is half the electrode alignment period, as shown in Figure 5. Here, the electrode alignment period refers to the center to center distance between two adjacent electrodes assuming that electrodes with the same width are placed at regular intervals, and this period is denoted as *h*. By stacking *S*1 and *S*2, virtual cells with a period (virtual electrode width) of *h*/2 are formed in the region where *S*1 and *S*2 are overlapped. The capacitance of each virtual cell can be calculated from the capacitances of the related cells of the two stacked sensor sheets. Because the basic structure of the proposed sensor sheet is the same as that of a traditional sensor sheet, the resolution can be increased by a factor of four without decreasing the measurement precision and flexibility. In addition, stacking three or more traditional sensor sheets is possible in principle, the resolution can be increased by a factor of *L*^2^ by stacking *L* identical sensor sheets with shifts in position of *h/L*.

### 3.2. Method of Calculating Pressure on Virtual Cells

**Figure 6.**
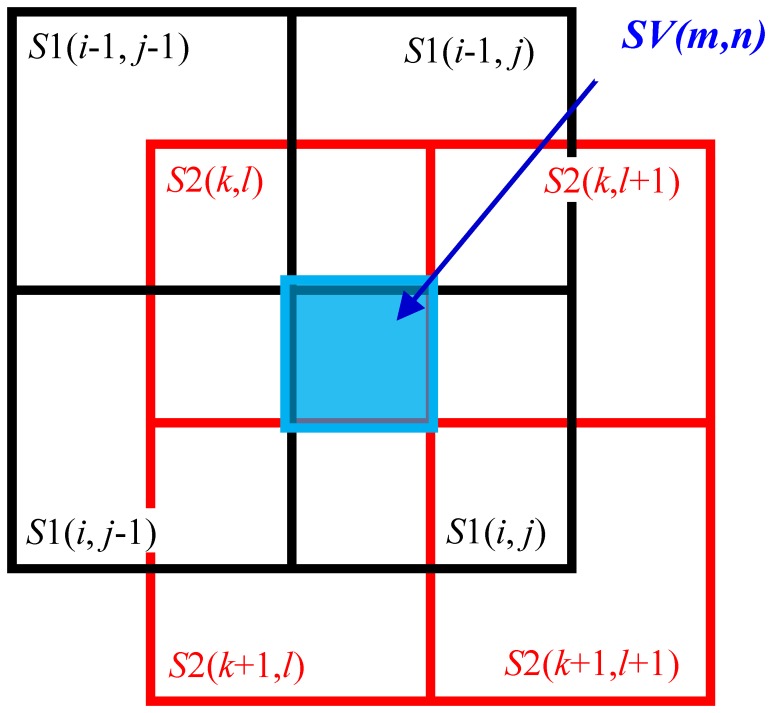
Virtual cells formed by stacking two identical sensor sheets.

The virtual cells formed by stacking *S*1 and *S*2 are denoted as *SV* (see Figure 6). The cell in the *i*th row and *j*th column of *S*1 is denoted as *S*1(*i,j*), the cell in the *k*th row and *l*th column of *S*2 is denoted as *S*2(*k,l*), and the virtual cell formed in the region where *S*1(*i,j*) and *S*2(*k,l*) overlap is denoted as *SV*(*m,n*). The capacitances of these cells are denoted as *C*_*S*1_(*i,j*), *C*_*S*2_(*i,j*), and *C_SV_*(*m,n*), respectively.

From Equations (1) and (4), the relationship between capacitance and pressure is given by Equation (14), which is applied to both *S*1 and *S*2. For sensor sheets with two or more layers, Equation (14) is applied to all the layers. The pressures corresponding to *C*_*S*1_(*i,j*) and *C*_*S*2_(*k,l*), which are denoted by *p*_*S*1_(*i,j*) and *p*_*S*2_(*k,l*), respectively, can be calculated using Equation (14). The pressure applied to the virtual cell *SV*(*m,n*), *i.e., p_SV_*(*m,n*), can be approximately obtained by calculating the average of *p*_*S*1_(*i,j*) and *p*_*S*2_(*k,l*), as expressed by Equation (15):

(14) $$p(i,j)=f^{-1}\left[d_{0}-{ɛ}_{0}{ɛ}_{r}\frac{S(i,j)}{C(i,j)}\right]$$

(15) $$p_{SV}(m,n)=\frac{1}{2}[p_{S1}(i,j)+p_{S2}(k,l)]$$

The relations between the subscripts *i,j,k,l* and *m,n* in Equation (15) is given by:

(16) $$\{\begin{array}{l} i=(m+1)\mathrm{mod}2+[(m+1)/2]\\ j=(n+1)\mathrm{mod}2+[(n+1)/2]\\ k=m\mathrm{mod}2+[m/2]\\ l=n\mathrm{mod}2+[n/2] \end{array}$$

where “mod” denotes modulo operation and “[ ]” denotes the Gauss' symbol.

The distribution of pressure can be obtained at a resolution fourfold higher than that of a traditional sensor sheet by processing as above. In this simple averaging using Equation (15), however, *p*_*S*1_(*i,j*) calculated from *C*_*S*1_(*i,j*) is the mean pressure applied to *S*1(*i,j*) and a non-uniform pressure distribution in the cell cannot be considered. This is the same for sensor sheet S2. Therefore, the following compensation process is performed to reduce the error caused by a non-uniform pressure distribution. As shown in Figure 6, the cell *S*1(*i,j*) of sensor sheet S1 overlaps with four cells of sensor sheet S2: *S*2(*k,l*), *S*2(*k*+2,*l*), *S*2(*k,l*+1), and *S*2(*k*+1,*l*+1), with each overlap comprising a quarter of the area of *S*1(*i,j*). The non-uniformity of the pressure distribution within *S*1(*i,j*) can be evaluated by comparing the outputs from the four cells. The compensated capacitance of *C*_*S*1_(*i,j*), $C_{S1}^{\prime }(i,j)$, is given by Equation (17).

Similarly, the cell *S*2(*i,j*) of sensor sheet S2 overlaps with four cells of sensor sheet S1: *S*1(*i,j*), *S*1(*i*-1,*j*), *S*1(*i,j*-1), and *S*1(*i*-1,*j*-1), with each overlap comprising a quarter of the area of *S*2(*k,l*), and the compensated capacitance of *C*_*S*2_(*k,l*), $C_{S2}^{\prime }(k,l)$, is given by Equation (18):

(17) $$C_{S1}^{\prime }(i,j)=\frac{4C_{S2}(k,l)}{C_{S2}(k,l)+C_{S2}(k+1,l)+C_{S2}(k,l+1)+C_{S2}(k+1,l+1)}C_{S1}(i,j)$$

(18) $$C_{S2}^{\prime }(k,l)=\frac{4C_{S1}(i,j)}{C_{S1}(i,j)+C_{S1}(i-1,j)+C_{S1}(i,j-1)+C_{S1}(i-1,j-1)}C_{S2}(k,l)$$

Calculating the corresponding pressures $p_{S1}^{\prime }(i,j)$ and $p_{S2}^{\prime }(k,l)$ from $C_{S1}^{\prime }(i,j)$ and $C_{S2}^{\prime }(k,l)$ given by Equations (17) and (18), respectively, we obtain Equation (19). Thus, the pressure applied to the virtual cell *SV* can be obtained:

(19) $$p_{SV}^{\prime }(m,n)=\frac{1}{2}[p_{S1}^{\prime }(i,j)+p_{S2}^{\prime }(k,l)]$$

## 4. Fabrication of the Prototype Sensor Sheet and Evaluation of Its Characteristics

### 4.1. Fabrication of the Prototype Sensor Sheet

**Figure 7.**
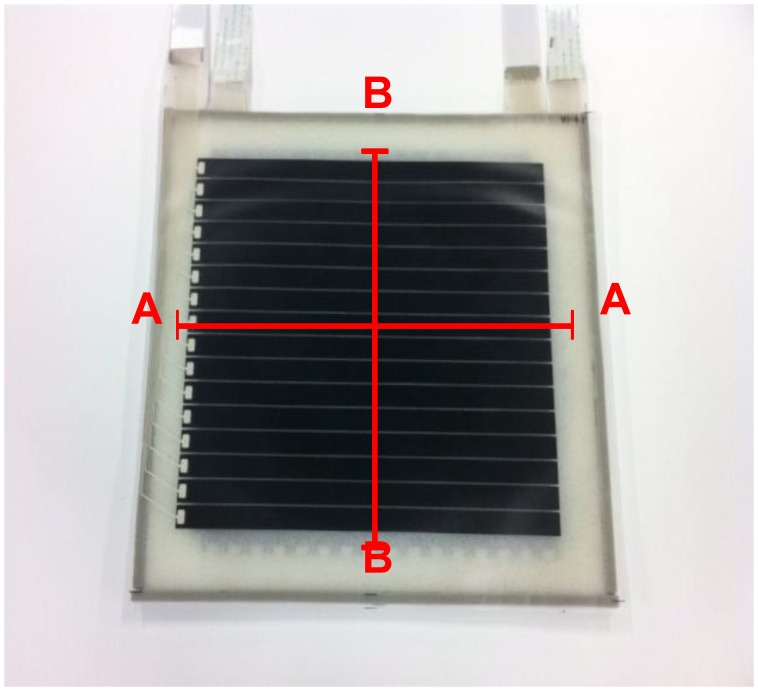
Picture of a prototype of the proposed sensor sheet.

**Figure 8.**
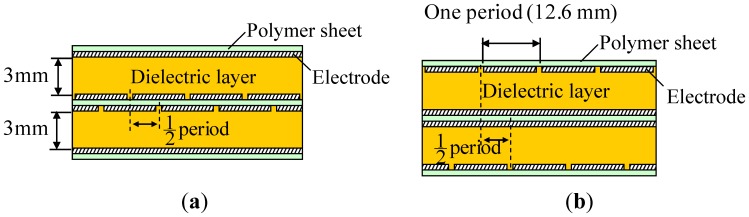
Cross sectional view of the two-ply sensor sheet shown in Figure 7. (**a**) A-A Section in Figure 7; and (**b**) B-B Section in Figure 7.

A prototype two-ply sensor sheet was fabricated to verify the effectiveness of the proposed structure. Figure 7 shows a picture of the prototype. Electrodes were formed by screen-printing conductive rubber ink onto polymer (urethane rubber) sheets. The number of electrodes per layer was 16, the electrode width was 11.6 mm, the gap between adjacent electrodes was 1 mm, so that the electrode alignment period was 12.6 mm. Because each of the electrode layers facing each other contains 16 electrodes, the number of cells formed in the sensor sheet was 16 × 16 = 256. Urethane foam of 3 mm thickness was used for the dielectric layer. Two such sensor sheets were fabricated and stacked with a shift of 6.3 mm, which is half the electrode alignment period, as shown in Figure 5. The two stacked sensor sheets have a common intermediate electrode sheet, a urethane rubber sheet having electrodes on both sides as shown by the cross sectional view in Figure 8. The structure was thus designed to limit the increase in the number of components due to the multilayered structure and to avoid relative movement between sensor sheets S1 and S2 while in use.

### 4.2. Evaluation of Sensor Characteristics

**Figure 9.**
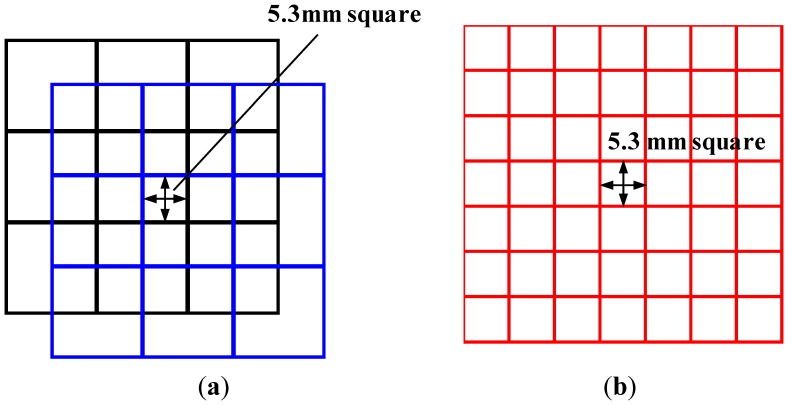
Conceptual diagram of the proposed sensor sheet (A in Table 1) and a traditional sensor sheet with the same resolution (B in Table 1). (**a**) A portion of the proposed two-ply sensor sheet A; and (**b**) A portion of sensor sheet B.

The characteristics of the proposed sensor were compared with those of traditional sensors to verify the effectiveness of the proposed multilayered structure. To this end, the following three sensors were fabricated and evaluated: A, the proposed two-ply sensor; B, a traditional sensor with high resolution (*i.e.*, with an electrode width about half that of A); and C, a traditional sensor corresponding to one of the two sheets used in A. For sensors A and C, the number of electrodes per layer was 16 and the electrode alignment period was 12.6 mm. Because of the two-ply structure, sensor A has a resolution corresponding to 6.3 mm square. To obtain this resolution using the traditional sensor, a sensor sheet containing thirty-two 5.3-mm-width electrodes per layer is required (see Figure 9b). Sensor B is a traditional sensor that satisfies these requirements. Table 1 gives a summary of the sensor parameters.

**Figure 10.**
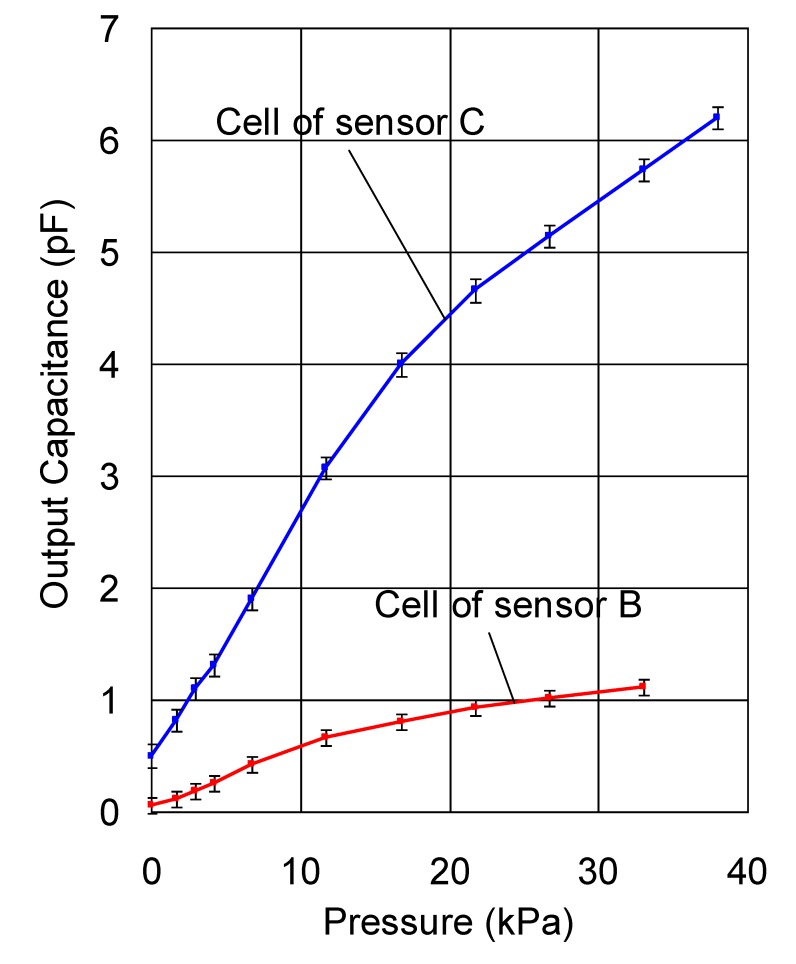
Relation between output (capacitance) and static pressure for the cells of sensors C and B.

Firstly, we compared the capacitance-pressure relationship and noise level of a cell of sensor C and that of sensor B. The mean output (capacitance) and its standard deviation when an uniform pressure was applied to a cell of sensor C and that of sensor B using stamps were plotted in Figure 10. The measurement was performed under the same ambient conditions and the pressure time was one minute. Although the hysteresis dependence of the response is not a topic of this paper, it should be noted that hysteresis cannot be completely removed because the sensor is made entirely of polymers. In the measurement carried out to obtain the result in Figure 10, the pressure was discretely increased at a long time interval to remove the influence of hysteresis.

Although the absolute noise of sensor C is larger than that of sensor B, the relative noise (ratio of the absolute noise to the mean output) of sensor C is much smaller than that of sensor B. As an example, the relative noise of sensor C under a uniform pressure of 22 kPa was 4%, while that of sensor B was 15%. The output (capacitance) of a cell under a certain pressure is proportional to its area and as a result, a cell with a smaller area is more easily affected by noise, leading to increased measurement error. The electric noise is believed to be coming from the wires, the circuit boards, as well as the sensor sheet itself.

The proposed two-ply structure (sensor A) has the same cell dimensions as sensor C. Since the calculation procedure described in Section 3 does not produce noise, we can say that the proposed structure has the same low noise level as sensor C, *i.e.*, the noise level of the proposed structure is lower than that of a traditional sensor sheet with the same resolution.

**Figure 11.**
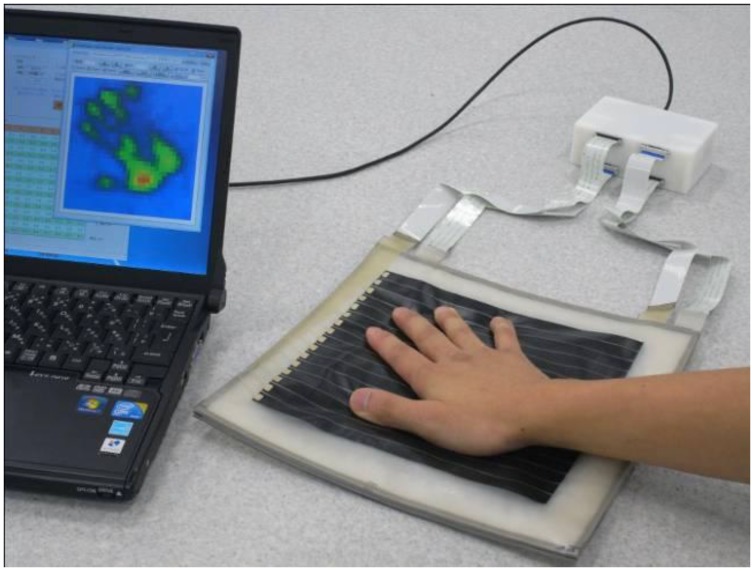
Picture when measuring the pressure of the palm of a hand.

**Figure 12.**
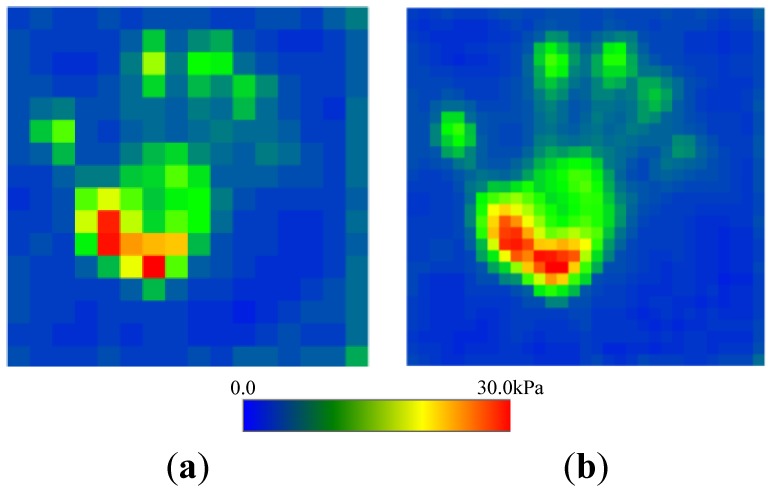
Distribution of pressure measured by pressing the palm of a hand onto the sensor sheet. (**a**) Traditional sensor sheet; and (**b**) Proposed two-ply sensor sheet.

Secondly, we confirmed the improvement on resolution by comparing the proposed two-ply sensor structure with sensor C. The distribution of pressure of the palm of a hand was measured (see Figures 11 and 12). Traditional sensor C, which has a low noise level, had a low resolution of 12.6 mm and was unable to image the shape of the palm clearly. In contrast, the proposed two-ply structure calculates the pressure with its fragmented 6.3-mm-square cells, obtaining a clearer image.

## 5. Conclusions

A multilayered capacitive tactile sensor sheet, which is made entirely of polymer materials such as rubber and urethane foam, was proposed to simultaneously achieve flexibility with high measurement precision and resolution, and its sensing principle was described. This multilayered sensor sheet can increase resolution fourfold or more, depending on the number of layers, without decreasing measurement precision. In addition, a prototype two-ply sensor sheet was fabricated and its characteristics were compared with those of traditional sensor sheets through actual measurements. It was confirmed that the proposed structure improves resolution without decreasing measurement precision and flexibility. Therefore, the measurement precision of the proposed structure is higher than that of a traditional sensor sheet with the same resolution. Here, when the region in which pressure is applied is smaller than a virtual cell, the measurement precision of the proposed structure is the same as that of a traditional sensor sheet with the same resolution.

The proposed two-ply sensor sheet can be installed on the surface of flexible objects, such as the soft exterior of human-interactive robots, because of its high flexibility. It is able to detect the pressure acting on the contact surface between flexible objects. In addition, since it has two independent sensor sheets and the measurement signals of the two sheets are independent, so even if one sensor sheet outputs an erroneous signal or breaks, the other sensor sheet is not affected, thereby increasing the reliability of a measurement system. A failure detection function can also be realized by incorporating logic to detect errors by comparing outputs from the overlapping cells.

## Figures and Tables

**Table 1.** Sensors used for performance comparison.

**Sensor**	**Sensor A (Proposed two-ply)**	**Sensor B (Traditional high resolution)**	**Sensor C (Traditional)**
**Number of electrodes**	16	32	16
**Width of electrodes**	11.6 mm	5.3 mm	11.6 mm
**Gap between two adjacent electrodes**	1.0 mm	1.0 mm	1.0 mm
**Resolution**	6.3 mm (virtual cell)	6.3 mm	12.6 mm

## References

[1] Mukai T., Kato Y. (2008). 1 ms soft areal tactile giving robots soft response. J. Robot. Mechatron..

[2] Herold B., Geyer M., Studman C.J. (2001). Fruit contact pressure distributions equipment. Comput. Electron. Agric..

[3] Zhang H., So E. (2002). Hybrid resistive tactile sensing. IEEE Trans. Syst. Man Cybern. Part B Cybern..

[4] Shimojo M., Namiki A., Ishikawa M., Makino M., Mabuchi K. (2004). A tactile sensor sheet using pressure conductive rubber with electrical-wires stitched method. IEEE Sens. J..

[5] Shimojo M., Araki T., Teshigawara S., Ming A., Ishikawa M. A Net-Structure Tactile Sensor Covering Free-Form Surface and Ensuring High-Speed Response.

[6] Kato Y., Hayakawa T., Mukai T. (2008). Soft areal tactile sensor using tomography algorithm. J. Robot. Mechatron..

[7] Takashima K., Horie S., Mukai T., Ishida K., Matsushige K. (2008). Piezoelectric properties of vinylidene fluoride oligomer for use in medical tactile sensor applications. Sens. Actuators A Phys..

[8] Sergio M., Manaresi N., Tartagni M., Guerrieri R., Canegallo R. (2002). A textile based capacitive pressure sensor. Proc. IEEE Sens..

[9] Lee H.-K., Chang S., Yoon E. (2006). A flexible polymer tactile sensor: Fabrication and modular expandability for large area deployment. J. Microelectromech. Syst..

[10] Mukai T., Hirano S., Nakashima H., Sakaida Y., Guo S. (2011). Realization and safety measures of patient transfer by nursing-care assistant robot RIBA with tactile sensors. J. Robot. Mechatron..

[11] Mukai T., Hirano S., Yoshida M., Nakashima H., Guo S., Hayakawa Y. Tactile-Based Motion Adjustment for the Nursing-Care Assistant Robot RIBA.

[12] Mukai T., Hirano S., Yoshida M., Nakashima H., Guo S., Hayakawa Y. Whole-Body Contact Manipulation Using Tactile Information for the Nursing-Care Assistant Robot RIBA.

[13] Sato S., Guo S., Inada S., Mukai T. (2012). Design of transfer motion and verification experiment of care assistant robot RIBA-II. Trans. Jpn. Soc. Mech. Eng. Ser. C..

[14] Cork R. (2007). XSENSOR technology: A pressure imaging overview. Sens. Rev..

